# Design considerations and analysis planning of a phase 2a proof of concept study in rheumatoid arthritis in the presence of possible non-monotonicity

**DOI:** 10.1186/s12874-017-0416-3

**Published:** 2017-10-02

**Authors:** Feng Liu, Stephen J. Walters, Steven A. Julious

**Affiliations:** 1 GlaxoSmithKline, Inc, 1250 South Collegeville Road, PO Box 5089, Collegeville, PA 19426-0989 USA; 2 Medical Statistics Group, University of Sheffield, Sheffield, UK

**Keywords:** *Emax*, NDLM, Dose response, Bayesian, Rheumatoid arthritis

## Abstract

**Background:**

It is important to quantify the dose response for a drug in phase 2a clinical trials so the optimal doses can then be selected for subsequent late phase trials. In a phase 2a clinical trial of new lead drug being developed for the treatment of rheumatoid arthritis (RA), a U-shaped dose response curve was observed. In the light of this result further research was undertaken to design an efficient phase 2a proof of concept (PoC) trial for a follow-on compound using the lessons learnt from the lead compound.

**Methods:**

The planned analysis for the Phase 2a trial for GSK123456 was a Bayesian *Emax* model which assumes the dose-response relationship follows a monotonic sigmoid “S” shaped curve. This model was found to be suboptimal to model the U-shaped dose response observed in the data from this trial and alternatives approaches were needed to be considered for the next compound for which a Normal dynamic linear model (NDLM) is proposed. This paper compares the statistical properties of the Bayesian *Emax* model and NDLM model and both models are evaluated using simulation in the context of adaptive Phase 2a PoC design under a variety of assumed dose response curves: linear, *Emax* model, U-shaped model, and flat response.

**Results:**

It is shown that the NDLM method is flexible and can handle a wide variety of dose-responses, including monotonic and non-monotonic relationships. In comparison to the NDLM model the *Emax* model excelled with higher probability of selecting ED90 and smaller average sample size, when the true dose response followed *Emax* like curve. In addition, the type I error, probability of incorrectly concluding a drug may work when it does not, is inflated with the Bayesian NDLM model in all scenarios which would represent a development risk to pharmaceutical company.

The bias, which is the difference between the estimated effect from the *Emax* and NDLM models and the simulated value, is comparable if the true dose response follows a placebo like curve, an *Emax* like curve, or log linear shape curve under fixed dose allocation, no adaptive allocation, half adaptive and adaptive scenarios. The bias though is significantly increased for the *Emax* model if the true dose response follows a U-shaped curve.

**Conclusions:**

In most cases the Bayesian *Emax* model works effectively and efficiently, with low bias and good probability of success in case of monotonic dose response. However, if there is a belief that the dose response could be non-monotonic then the NDLM is the superior model to assess the dose response.

**Electronic supplementary material:**

The online version of this article (10.1186/s12874-017-0416-3) contains supplementary material, which is available to authorized users.

## Background

An ongoing and serious challenge facing the pharmaceutical industry is the high failure rate in the late phase of drug development [1]. It has been reported that approximately 50% of Phase 3 clinical trials fail and the main explanations are the wrong dose being selected or poor understanding of the dose response in Phase 2 trials [1, 2]. Therefore, it is critical to identify the correct dose in Phase 2 clinical trials to improve the Phase 3 success rate and thus increase research and development productivity [3, 4].

An assessment of dose response normally starts with a linear or nonlinear regression of a drug response for given doses [5]. Many biological activities follow a 4-parameter logistic model, and the *Emax* model is a special case of the 4-parameter logistic model [3]. Among the possible dose response models, *Emax* model is one of the most widely applied models relating drug concentrations to effects [3]. In practice, the *Emax* model assumes the drug effect is proportional to the dose, i.e. the bigger the dose, the bigger the effect. Thomas et al. [6] showed that majority of dose response models in the dose response of small molecule compounds were *Emax* models based on dose response curves from a single company and there were two cases reported a likely U-shaped dose response that *Emax* model failed to fit [6].

As the name implies a U-shaped dose response is a dose response where there is a down-turn of the clinical dose-response relationship at higher doses. In the context of the problem being investigated, we had a prior belief from a lead compound, where a U-shaped dose response was observed, that the dose response for the follow-on compound in the same drug class may also be U-shaped. For this reason, a U-shaped dose-response is considered to be pharmacologically plausible for the follow-on compound as well as for the reason that a U-shaped dose response had been seen in other biological treatments for RA [7–10].

There are a number of dose response models available to handle the non-monotonic U-shaped dose response relationships [4]. One alternative is the Normal Dynamic Linear Model (NDLM) which originated in time series modelling and is a method for model smoothing using information borrowed from neighbouring doses [11]. Berry [12] then proposed the NDLM model for the adaptive designs and in the post-herpetic neuralgia trial, Smith et al. [13] applied a Bayesian NDLM model to a pharmaceutical drug trial where patients were randomised to a dose based on the dose response model estimated from a posterior distribution. A Bayesian NDLM model was also used in an Acute Stroke Therapy by Inhibition of Neutrophils (ASTIN) trial [14]. In the ASTIN trial patients were allocated 1 of 15 doses, or a placebo, adaptively based on the response and the study allowed for early termination for efficacy or futility based on posterior probability using a Bayesian NDLM model. In the ASTIN trial, a Markov chain Monte Carlo approach was used to derive a posterior distribution for the model parameters which informed the estimation of the ED95. In addition, there have been other applications of NDLM model such as in in phase 2/3 study for dose selection of diabetes drug development [15].

For the study being planned there was an interest in the comparisons of both *Emax* model and NDLM models for the dose response assessment in a Phase 2a trial in patients with rheumatoid arthritis (RA). The Phase 2a trial was initially designed to investigate the treatment effect of different dose levels of GSK123456, using Bayesian *Emax* model which was used to guide the Bayesian analysis in searching for the dose levels targeting at ED90 for future cohorts. The compound later failed since a U-shaped like curve was observed in the dose response. The *Emax* model makes an assumption of a monotonic dose response relationship which was seemed to be violated in this trial.

A follow-on compound GSK654321, which is in the same drug class of GSK123456, is in development. The chance for GSK654321 having a U-shaped curve cannot be ruled out, therefore the emphasis of this manuscript is to find a suitable dose response model and design for future Phase 2a design of GSK654321, which would provide reasonable design operating characteristics under both monotonic dose response and non-monotonic dose response. In the following section, two main statistical models (*Emax* and NDLM) for estimating a dose response relationship are described and compared in a Phase 2a trial in patients with rheumatoid arthritis (RA). Also to use extensive simulations to show how the two models perform under a fixed and adaptive designs under a variety of assumed dose-response profiles with a focus on U-shaped response curve, a pharmacologically plausible dose response curve in GSK654321.

## Methods

### Background of clinical trials in RA patients

A primary endpoint of a typical Phase 2 clinical trial is the change from baseline in DAS28 score. DAS28 is a measure of disease activity score and the number 28 refers to the 28 joints that are examined in this assessment.

To calculate the DAS28 [16], a clinician will:

1. count the number of swollen joints (out of the 28);
2. count the number of tender joints (out of the 28);
3. take blood to measure the erythrocyte sedimentation rate (ESR) or C reactive protein (CRP);
4. ask the patient to make a ‘global assessment of health’ (indicated by marking a 10 cm line between very good and very bad).

The results from these four domains are then combined to produce an overall disease activity score ranging from 2 to 10, with a higher score indicating more disease activity. A DAS28 of greater than 5.1 implies active disease, less than 3.2 low disease activities, and less than 2.6 as remission.

### Bayesian *Emax* model

The *Emax* model is a widely applied model relating drug concentrations to effects [3] and was planned for the analysis of dose response in the Phase 2a trial.

The *Emax* model is written as

1 $$ \Delta \mathrm{DAS}28=\mathrm{E}0+\frac{Ema{x}^{\ast } Dose}{\mathrm{ED}50+ Dose}+\varepsilon, \varepsilon \sim \mathrm{N}\left(0,{\sigma}^2\right) $$

where ΔDAS28 is the change in DAS28 score from baseline at day 56 post-randomisation, E0 is the basal effect corresponding to the response when the drug dose is equal to 0, *Emax* is the maximum achievable increase or decrease over placebo response, ED50 is the dose which produces 50% of the effect. All the doses were half-log spaced at design stage with exception of 20 mg/kg. The maximum dose level across the study cohorts is 30 mg/kg. The 30 mg/kg dose is the maximum tolerated dose for the study based on prior studies. If the posterior mean of ED90 exceeded 30 mg/kg, the maximum planned dose of 30 mg/kg is used. The priors of model parameters *E0* and *Emax* follow a Normal distribution with large variance i.e. N(0,1E4) and the prior distribution of ED50 are N(3,1E2). The prior on σ^2^ is an inverse gamma distribution (IG(0.5,0.7). Markov Chain Monte Carlo (MCMC) were used to simulate the posteriors distribution: 2500 samples were used to estimate the model parameter after burn-in of 500. A larger burn-in was run and didn’t significantly improve the model fitting and estimation parameters.

The parameters of interest for the *Emax* model can be estimated by maximum likelihood estimation (MLE) and Bayesian methods – we chose this approach as Bayesian statistics [17] integrates information into the computation of the posterior probability of parameters, using the accumulated data observed so far for later doses and prior information for the early doses. In addition, the parameters from Bayesian method are displayed as distributional profile - which can be useful to illustrate uncertainty - and offer a robust estimation of parameters in complicated model [17].

### Bayesian normal dynamic linear model (NDLM)

A NDLM can be used to fit to estimate the dose-response relationship. The description of the NDLM used in the analysis is shown below,

2 $$ \Delta \mathrm{DAS}{28}_{\mathrm{j}\mathrm{k}}\sim \mathrm{N}\left({\theta}_{\mathrm{j}},{\sigma}^2\right), $$

$$ {\theta}_{\mathrm{j}}={\theta}_{\mathrm{j}-1}+{\updelta}_{\mathrm{j}-1}+{\omega}_{\mathrm{j}};\mathrm{where}\kern0.3em \mathrm{j}=2;{\omega}_{\mathrm{j}}\kern0.3em \sim \kern0.3em \mathrm{N}\left(0,{\sigma_{\theta}}^2\right),{\updelta}_{\mathrm{j}}={\updelta}_{\mathrm{j}-1}+{v}_{\mathrm{j}};\mathrm{where}\kern0.3em {v}_{\mathrm{j}}\kern0.3em \sim \kern0.3em \mathrm{N}\left(0,{\sigma_{\updelta}}^2\right) $$

where ΔDAS28 is the observed individual change in DAS28 score from baseline at day 56 post-randomisation at Dose_j_. The likelihood of DAS28 at day 56 change from baseline follows a Normal distribution with mean (θ_j_) for each Dose_j_ and with variance of σ^2^, the Dose_j_ is assumed to be spaced equally. θ_j_ is the estimated treatment effect at Dose_j_. Furthermore, θ_j_ has a linear relationship with neighboring θ_j−1_ with intercept θ_j−1_ and slope of δ_j−1_. θ_1_ is the untreated or placebo response when the drug dose is equal to 0 and both θ_1_ and δ_1_ follow Normal distributions. Similar to *Emax* model, the coefficients for the NDLM model can be estimated from maximum likelihood methods [18] and Bayesian methods – we used the Bayesian NDLM method because Bayesian methods offer robust estimation of parameters with complicated models and provides better model fitting in both monotonic and non-monotonic dose response [17].

The prior distribution on θ has a vague Normal distribution with a large variance estimated from inverse-gamma distribution (IG(0.5, 72). The prior distributions on σ^2^ and the evoluation variance σ_θ_^2^ and σ_δ_^2^ are inverse-gamma distribution (IG(0.5, 72).

### Motivating study

**Fig. 1:**
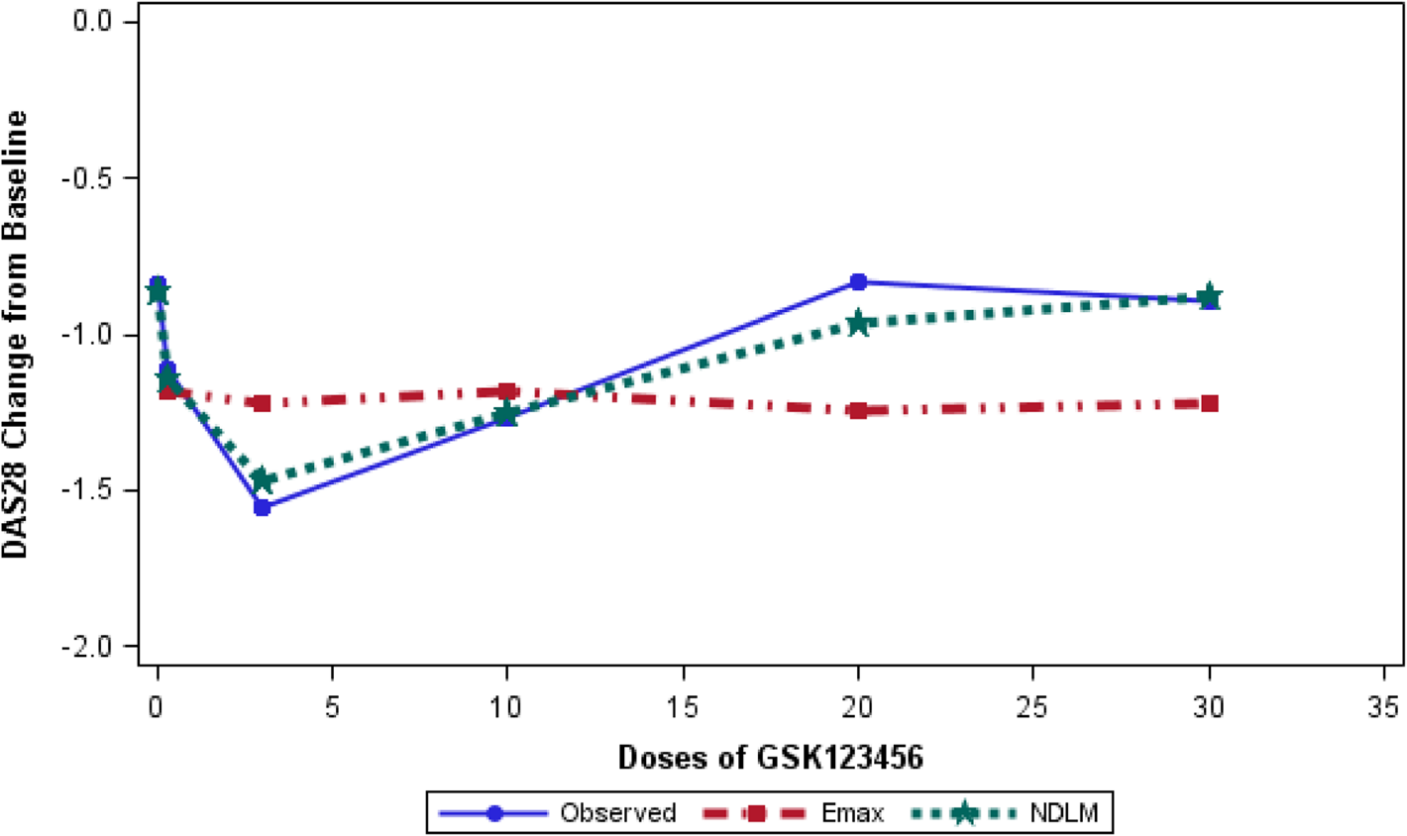
Mean and estimated dose response of mean change in DAS28 scores from baseline ﻿using Bayesian *Emax* and NDLM models. The mean changes in DAS28 score between the doses were connected with a straight line in solid blue lines; the data are for illustration purpose so the error bars are not presented. *Emax* model ﻿is displayed ﻿as red dash/dotted line﻿ ﻿and NDLM model ﻿as green dotted line﻿

An initial Phase 2a Proof-of-concept (PoC) study was undertaken to demonstrate whether a new drug, GSK123456, achieves a certain level of pre-designated efficacy at a planned dose in RA [19]. The first part (Part A) of this PoC study was a learning phase with single dose escalation using a cohort randomised trial [19]. Patients were randomised within each cohort to either placebo or an active dose of GSK123456. Only the starting dose in cohort 1 was pre-defined and subsequent doses for other cohorts were selected using a Bayesian dose response *Emax* model [19]. A U-shaped dose response curve for DAS28 change from baseline was observed with the highest response at 3 mg/kg (Fig. 1). A consequence of this was the estimation of ED90 was suboptimal with higher variability.

A dose response in a new class of compound or target is generally unknown due to the biology and is not well understood, especially the drug is never being tested in healthy volunteers or patients. Further pharmacokinetic and pharmacodynamics data suggests the U-shaped curve may be due to moderate binding affinity and rapid off-rate of GSK123456 as compared to the higher affinity OSM receptor causing a protein carrier effect [19].

The follow-on compound GSK654321 is in the same drug class. It binds to the same binding site as GSK123456 and it is believed to have therapeutic properties but with higher potency. Therefore, the chance of U-shaped dose response cannot be ruled out. It is important to highlight however that *Emax* was the pre-specified analysis. Given the U-shaped curves being pharmacologically plausible in the follow-on compound GSK654321, there is a strong desire to compare and adopt a more flexible model, such as NDLM model, to handle both monotonic and non-monotonic dose response in the design and analysis consideration.

We have observed how *Emax* model was suboptimal in modelling the dose response. We then applied a model – NDLM – retrospectively, we know should work for the observed data and then demonstrated it was superior. For NDLM to be prospectively planned for GSK654321 there is need first to do further evaluations of its properties in the context of a RA PoC study design in the possible presence of non-monotonicity.

In next sections, we will explore the NDLM model, to compare the performance of the *Emax* model and the NDLM under various assumptions about the shape of the dose response curves - flat curve, *Emax* like curve, Log-linear curve and U-shaped curve.

## Simulation

### Dose response models in the evaluation

**Fig. 2:**
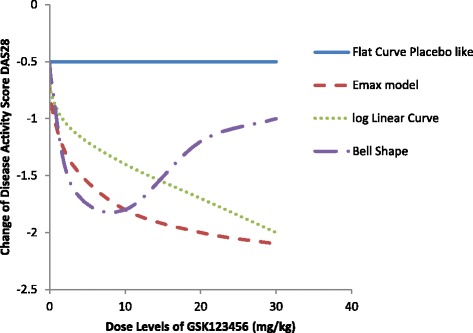
The four true Dose-response profiles used in the simulations. The model profiles include a placebo like flat curve which is denoted in blue and is fixed at −0.5 for all dose levels, a dose proportional *Emax* model in red, a log-linear model in green, and a U-Shaped model in purple. The label for the vertical axis is the change in DAS28 score from predose at day 56 post-randomisation

For the simulations four true dose response profiles (Fig. 2) are used for the primary endpoint, change in DAS28 score from baseline to day 56, to mimic the wide range of dose response scenarios likely to be observed and be analysed as dose response methods in clinical practice. In all models, the placebo effect (on the background of MTX) was set to be −0.5. That is a change in DAS28 score from baseline to day 56 post-randomisation of −0.5 points i.e. a small decline/improvement is disease activity. The error term ε was assumed to be independently Normally distributed with a mean of 0 and a variance of 1.44 for *Emax*, Log linear and U-shaped curve, which was the estimated variance from PoC study of GSK123456 (Fig. 2), the error term has variance 0.25 for placebo like response.

**Table:** 

Profile 1	Flat curve: ΔDAS28 = −0.5 + ε
Profile 2	*Emax* curve:y ΔDAS28 = −0.5–1.7*Dose/(2.5 + Dose) + ε, ED50 is 2.5.
Profile 3	Log linear curve: ΔDAS28 = −0.5 -log(Dose + 1) + ε
Profile 4	U-shaped curve: ΔDAS28 follows a predefined U shaped curve with: ΔDAS28 = (−0.5,-0.7,-1.6,-1.8,-1.2,-1, −0.6) for dose 0, 0.03, 0.3, 3, 10, 20, and 30 mg/kg respectively.

These four profiles were chosen as plausible dose responses for the new compound in development GSK654321 ranging from a null effect (Profile 1) to what was previously observed with GSK123456 (Profile 4).

The scenarios of fixed design simulation and adaptive design simulation are discussed in the next section. The two basic designs set up are a fixed design and an adaptive design. The fixed design assumes that all six doses and placebo are allocated to a fixed number of patients. No adaptations are adopted in this design. In the adaptive design, the subjects are allocated according to the dose responses of all the subjects enrolled in the study.

### Design of the Simulation Study

The range of doses is between 0.03 mg/kg to 30 mg/kg. The design is a parallel design and the total target sample size is 64. The goal of the trial is to characterise the dose-response curve at various doses. The fixed design assumes that all 6 doses are allocated to a fixed number of patients with no interim analysis or adaptation of the dose. In the adaptive simulation, the subjects are allocated due to the subjects’ response in the study at the end of each cohort.

Decisions regarding success and futility of the trial at completion are made based on the probability of DAS28 relative to control greater than clinically significant difference (a decrease of 0.95 as measured by DAS28 change from baseline between placebo and treatment). The positive difference of placebo and treatment is used to facilitate the positive effect and probability calculation. All the designs except fixed scenarios include 8 cohorts, with 8 patients in each cohort (2 on placebo and 6 on active treatment).

An adaptive design was used in the PoC design of GSK123456 and is considered as a better option than fixed design since it increases the chance of stopping a failed compound and expediting a good one as well as potentially maximizing the information on the doses which are most interest to carry forward for later development. For GSK654321 the study design has not been finalised. The wish therefore was to evaluate modelling the dose response using NDLM or *Emax* for different options for the study design which we have detailed. The follow-on compound GSK654321 is in the same drug class as GSK123456 which demonstrated good safety and tolerability in the PoC study [19], so there is no single dose escalation planned for the PoC study in GSK654321.

ED90 is defined as the dose to achieve 90% of maximum DAS28 response with the lower dose chosen if there are multiple values. In this calculation the maximum response is estimated from the maximal DAS28 effect at all doses. The 90% (ED90) of maximum response is then calculated as the lowest nominal dose in which is closest to the estimated dose that achieves 90% of maximal efficacy. The following fixed design as well as adaptive design scenarios are considered in the design options and evaluations.

**Table:** 

Scenario 1	Fixed design; the design is non-adaptive, the study allocates 8 patients to receive doses of GAK654321 (0.03, 0.3, 3, 10, 20 and 30 mg/kg) and 16 patients to receive placebo. There is no interim stopping and adaptation in the fixed design. The evaluation of final success will occur at the end of the study.
Scenario 2	No adaptive allocation; the ratio of patients (100% of the planned sample size) randomized into each study dose (placebo, 0.03, 0.3, 3, 10, 20 and 30 mg/kg) are 2:1:1:1:1:1:1. The placebo is given to a fixed proportion of the sample size allocation to ensure there is enough power for treatment comparisons vs. placebo. There are a total of 8 cohorts (6 treated +2 placebo) and the interim analysis will occur between cohorts, for example, at 8 patients, 16 patients, 24 patients, 32 patients (50%), 40 patients (62.5%) and 48 patients (75%) enrolled and complete the primary endpoint assessment (day 56 post-randomisation DAS28 score). The study is evaluated with the interim study success and interim study futility.
Scenario 3	Half adaptive, the first 50% of subjects are fixed allocated using pre-defined allocation ratio of treatments and placebo followed by adaptive allocation for the rest of the subjects based on the posterior distribution of dose around ED90; the placebo is given to a fixed proportion of the sample size allocation to ensure we have enough power for treatment comparisons vs. placebo. The fixed proportion is 25% of the total sample size. For each study dose (0.03, 0.3, 3, 10, 20 and 30 mg/kg), the 4 patients (50% of the planned sample size) will be randomized first, prior to any interim analysis. The dose response curve will then be fitted using the dose response model and ED90 is estimated. For each subject randomised into the study afterwards, the dose level will be randomized to the dose close to the ED90 dose response. The interim analysis will occur at 32 patients (50%), 40 patients (62.5%) and 48 patients (75%) that complete the primary endpoint assessment. The study is evaluated for interim study success and interim study futility.
Scenario 4	Adaptive allocation after the first cohort. In the fully adaptive simulation, the placebo is given a fixed proportion of the sample size allocation to ensure there is enough power for treatment comparisons vs. placebo. The fixed proportion is 25% of the total sample size. The dose response curve will be fitted using the dose response model and ED90 is estimated. For each subject randomised into the study afterwards, the dose level will be randomized to the dose close to ED90 dose response. The interim analysis will occur between cohorts, for example, at 8 patients, 16 patients, 24 patients, 32 patients (50%), 40 patients (62.5%) and 48 patients (75%) enrolled and complete the primary endpoint assessment. The study is evaluated for interim study success and interim study futility.

The simulation and analysis are performed using a data simulation and analysis software - FACTs (Fixed and Adaptive Clinical Trial Simulator) version 2.1 and 4.05 developed by Tessella and Berry Consultant. Simulated data are fitted using similar *Emax* model and NDLM models as described in Eqs. 1 and 2. It is possible that the choice of informative prior impacts the simulation results ﻿[20]﻿, for consistency and comparison purpose, a vague prior is chosen in the calculation and simulation. The priors for the *Emax* model parameters *Eo* and *Emax* are vague and follow a Normal distribution with large variance. Thus, the prior of model parameter *E*_0_ is N(0,1E4) and the prior distribution of ED50 is N(3,1E2). The vague prior distribution of evolution variance for NDLM model is inverse-gamma distribution (IG(0.5, 72). Additionally, selected informative priors are explored in the simulations. The simulation starts with fixed seed and all results are based on 5000 simulations. The number of simulations and number of MCMC simulations as 2500 with burn-in of 500 are chosen based on the estimated minimum precision.

### Decision criteria in adaptive design simulation

Decision criteria for interim success, interim futility, final success and final futility in the adaptive design simulation are displayed in Table 1. For the fixed design, the final success is based on at least 95% posterior probability that the d_ED90_ dose achieves a drug effect greater than the control or placebo, otherwise it is final futility. For all other adaptive design (scenario 2, 3, and 4), the decision criteria of the interim success, interim futility, final success and futility are presented in Table 1.

**Table 1:** Decision criteria at the interim analysis and final analysis in the proposed design scenarios

Decision Criteria	Interim Success	Interim Futility	Final Success	Final Futility
Pr(|R_ED90_ –Ctrl| > 0)	>95%	<20%		
Pr(|R_ED90_ –Ctrl| > 0.95)	>70%			
Pr(|Rd_max_ –Ctrl| > 0) > 0.95 and Pr(|R_ED90_ –Ctrl| > 0) >95%			Yes	No

Pr(|R_ED90_ –Ctrl| > 0): The probability of dose response near ED90 dose level achieves a drug effect greater than the control or placebo

Pr(|R_ED90_ –Ctrl| > 0.95): The probability of dose response near ED90 dose level achieves a drug effect greater than the control or placebo and 0.95 is the clinical significant difference

Pr(|Rd_max_ –Ctrl| > 0): The probability of any dose with maximal effect achieves a drug effect greater than the control or placebo

Only final success and futility are accessed in fixed design

When there is truly is no effect or a placebo like effect, the Type I error rate is calculated based on the chance of rejecting the null hypothesis (when it is true). In the context of this simulation it would also be the chance of incorrectly accepting that the drug has a dose response, the false positive rate, and the statistical bias.

## Results

### Design comparisons using simulation

The results from the simulations giving the probability of interim and final success and failure in fixed design (S1), no adaptive allocation (S2), half-adaptive (S3) and fully adaptive (S4) using Bayesian *Emax* model and NDLM model are displayed in Table 2.

**Table 2:** Probability of success and failures at interim and final analysis at fixed and adaptive design scenarios

True Dose Response	Design Scenarios	Models Comparisons	Early success	Early failure	Final success	Final failure	Total Success	Mean subjects
Placebo like flat curve	Fixed Design (S1)	Bayesian *Emax*	–	–	0.06	0.94	0.06	64
		Bayesian NDLM	–	–	0.17	0.83	0.17	64
	No Adaptive design (S2)	Bayesian *Emax*	0.00	0.39	0.04	0.57	0.04	51
		Bayesian NDLM	0.03	0.03	0.16	0.78	0.18	58
	Half Adaptive design (S3)	Bayesian *Emax*	0.00	0.23	0.04	0.73	0.04	61
		Bayesian NDLM	0.00	0.00	0.12	0.88	0.12	64
	Adaptive (S4)	Bayesian *Emax*	0.03	0.39	0.04	0.54	0.07	55
		Bayesian NDLM	0.01	0.01	0.12	0.86	0.13	64
*Emax* curve	Fixed Design (S1)	Bayesian *Emax*	–	–	0.98	0.02	0.98	64
		Bayesian NDLM	–	–	0.98	0.02	0.98	64
	No Adaptive design (S2)	Bayesian *Emax*	0.74	0.03	0.19	0.04	0.93	38
		Bayesian NDLM	0.82	0.01	0.09	0.08	0.91	37
	Half Adaptive design (S3)	Bayesian *Emax*	0.74	0.00	0.26	0.01	0.99	55
		Bayesian NDLM	0.53	0.00	0.44	0.03	0.97	57
	Adaptive (S4)	Bayesian *Emax*	0.80	0.03	0.15	0.02	0.95	42
		Bayesian NDLM	0.55	0.00	0.41	0.04	0.96	56
Log Linear Curve	Fixed Design (S1)	Bayesian *Emax*	–	–	0.96	0.04	0.96	64
		Bayesian NDLM	–	–	0.95	0.05	0.95	64
	No Adaptive design (S2)	Bayesian *Emax*	0.64	0.05	0.24	0.08	0.88	40
		Bayesian NDLM	0.74	0.00	0.15	0.11	0.89	40
	Half Adaptive design (S3)	Bayesian *Emax*	0.58	0.00	0.40	0.02	0.98	57
		Bayesian NDLM	0.45	0.00	0.47	0.08	0.92	58
	Adaptive (S4)	Bayesian *Emax*	0.70	0.03	0.24	0.03	0.94	45
		Bayesian NDLM	0.51	0.00	0.43	0.06	0.94	56
U-Shaped curve	Fixed Design (S1)	Bayesian *Emax*	–	–	0.26	0.74	0.26	64
		Bayesian NDLM	–	–	0.92	0.08	0.92	64
	No Adaptive design (S2)	Bayesian *Emax*	0.10	0.26	0.14	0.50	0.24	47
		Bayesian NDLM	0.63	0.01	0.17	0.18	0.80	43
	Half Adaptive design (S3)	Bayesian *Emax*	0.06	0.09	0.10	0.75	0.16	62
		Bayesian NDLM	0.41	0.00	0.47	0.12	0.88	59
	Adaptive (S4)	Bayesian *Emax*	0.14	0.24	0.10	0.52	0.24	54
		Bayesian NDLM	0.42	0.00	0.44	0.14	0.86	58

For *Emax* like true dose response, the total probability of success is 98% and 98% in fixed design; 93% vs. 91% in No-Adaptive Allocation design, 99% vs. 97% under Half Adaptive scenario and 95% vs. 96% under Adaptive Allocation scenario for *Emax* and NDLM models respectively. The average sample sizes in the trials are less in the No-Adaptive Allocation design, half adaptive and adaptive design than fixed design. Similar results and trends are also shown for log linear dose response curve.

The Type I error is inflated in Bayesian NDLM model in all scenarios under the current prior. The higher Type I error could potentially lead to a false investment decision and further work when a compound does not truly have an effect. Though the inflation of type I error rate is not a regulatory risk for a Phase 2a study it is a potential risk to the sponsor. The Phase 2a study is still an investigative study so the consequences risks are less and once the final study design is established the simulations will need to be reinvestigated with the decision criteria (as described in Table 1) set so the Type I error is controlled.

Table 3 displays the additional operating characteristics of the model fitting to the data that were analysed using the *Emax* model and NDLM model for Half Adaptive (S3) design. The proportion of times the dose being selected as ED90 are displayed with each of the four curves. The ED90 of the true *Emax* curve is likely to be between 20 and 30 mg/kg. Similar results for No adaptive (S2) and fully adaptive (S4) are presented in Additional file 1: Table S1 and Table S2 respectively.

**Table 3:** Proportion of doses being selected as ED90 of Bayesian *Emax* and NDLM model at different dose response curves in the Half Adaptive design settings (Scenario 3)

	Dose Level (mg/kg)					
	0.03	0.3	3	10	20	30
Bayesian *Emax* Model						
Proportion of doses being selected as ED90						
Flat placebo like Curve^a^	0%	0%	0%	0%	38%	0%
*Emax* like Curve	0%	0%	0%	0%	89%	11%
Log Linear Curve	0%	0%	0%	0%	81%	19%
U-Shape Curve^a^	0%	0%	0%	0%	64%	0%
Bayesian NDLM Model						
Proportion of doses being selected as ED90						
Flat placebo like curve	16%	17%	14%	14%	11%	12%
*Emax* like Curve	1%	1%	14%	25%	35%	26%
Log Linear Curve	1%	1%	5%	11%	29%	54%
U-Shape Curve	2%	42%	48%	5%	2%	0%

^a^ED90 is missing where the maximum dose was not estimated correctly

Results from the simulations show that the Bayesian *Emax* model is able to find the correct dose for ED90 almost 100% of time (proportion of ED90 as 20 and 30 mg/kg) when the true response is either an *Emax* curve or log linear curve, comparing to approximately 6﻿1﻿%–83% using Bayesian NDLM model. If the true dose response relationship is assumed to follow a U-shaped curve, the proportion of simulations selecting the ED90 as 0.3 and 3 mg/kg are 0% vs 82% in non-adaptive design, 0% vs 90% in Half-adaptive setting and 0% vs 91% in Adaptive setting using *Emax* model and NDLM model respectively when the true ED90 is around 2.5 mg/kg. NDLM is able to identify the correct ED90 doses 58% or 76% of the time when the true response is an *Emax* or log linear curve respectively.

All the simulated results seem to indicate that the *Emax* model performs better when the dose responses are monotonic and the NDLM model is a more robust approach in all four types of model and is superior to identify the correct ED90 doses when the true response followed a U−shaped curve.

**Fig. 3:**
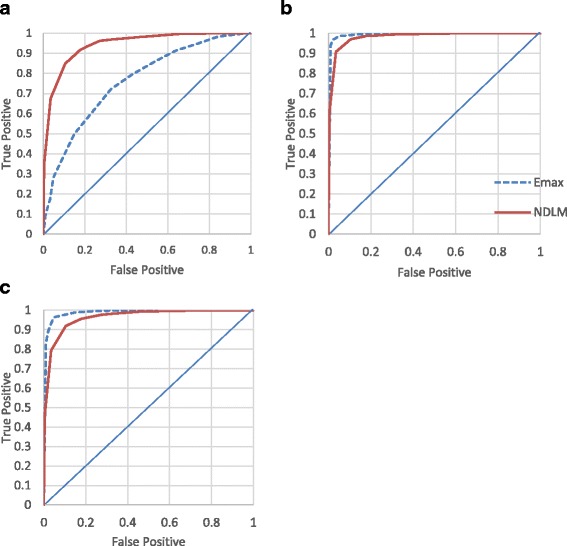
Receiver operating characteristic ROC curve display the true positive rate (statistical power) and false positive rate for Bayesian *Emax* (red) and NDLM model (blue) under Fixed design (S1). Bayesian *Emax* (blue dashed line) and NDLM model (red solid line) and dose response following **a** U-Shaped, **b** *Emax* or **c** Loglinear curve

**Fig. 4:**
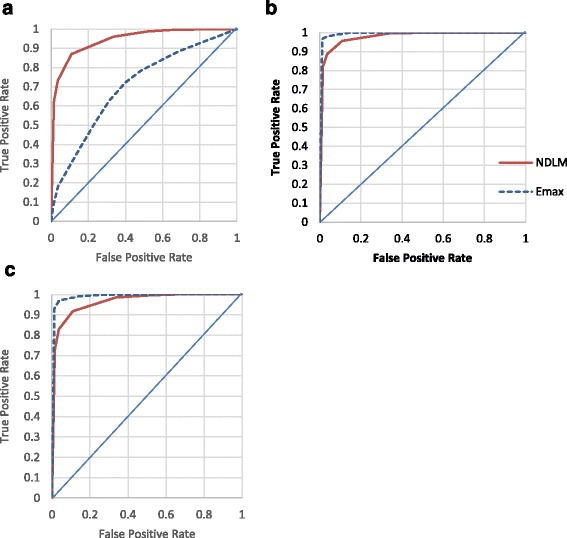
Receiver operating characteristic ROC curves display the true positive rate (statistical power) and false positive rate for Bayesian *Emax* (red) and NDLM model (blue) under Half adaptive design (S3). Bayesian *Emax* (blue dashed line) and NDLM model (red solid line) and dose response following **a** U-Shaped, **b** *Emax* or **c** Loglinear curve

In earlier comparisons of the *Emax* and NDLM models, the same decision rules were applied and to assess the type I errors. To facilitate for a fair comparison of power without the need for recalibrating type I error at each design, Receiver Operating Characteristic curves (ROC curve) for the fixed design (S1) and half-adaptive (S3) are presented in Figs. 3 and 4 respectively. The ROC curves draw a plot of the true positive rate against the false positive rate for the different possible decision criteria. Since any increase in sensitivity is accompanied by a decrease in specificity, the ROC shows the tradeoff between sensitivity and specificity. For each design, the true positive rates from Bayesian *Emax* and NDLM model at assumed U-shaped, *Emax* or Loglinear curves are plotted against the corresponding false positive rates from flat curve. The closer the curve follows the left border and the top border of the ROC space, it shows the better sensitivity given specificity. Similar ROC curves for non-adaptive (S2) and adaptive (S4) design are presented in supplemental material.

Under half adaptive design, the ROC curve of Bayesian *Emax* model is closer to the left and top borders than NDLM model when the assumed curves follow *Emax* or loglinear curves, so *Emax* model performs better. When the type I error rate is at 5%, the true positive rate of to Bayesain *Emax* model is approximately at 97% for both *Emax* curve and loglinear curve and the true positive rate is 90% and 85% for both *Emax* curve and loglinear curve using NDLM model. For U-shaped curve, the Bayesian NDLM model performed better than *Emax* model. The results are in line with earlier conclusion that *Emax* model outperforms if dose response is monotonical and NDLM model is better when the dose response is U-shaped.

### Assessment of bias

The assessment of statistical bias through simulation at each dose level (placebo, 0.03, 0.3, 3, 10, 20, and 30 mg/kg) is calculated as the difference in the estimated mean response using *Emax* or NDLM models against the assumed true response profile (at each dose level). The difference from the true dose response profile is estimated for each simulation. The mean difference - and bias - is taken as the mean difference for the dose response from the truth across all 5000 simulations.

The Bayesian *Emax* model is compared to the NDLM model under four profiles of true dose response being *Emax* curve (Fig. 3a), flat curve (Fig. 3b), log linear curve (Fig. 3c), and U-shaped curve (Fig. 3d) for each of the four design scenarios: fixed design, no adaptive (S2), half adaptive (S3) and fully adaptive (S4).

**Fig. 5:**
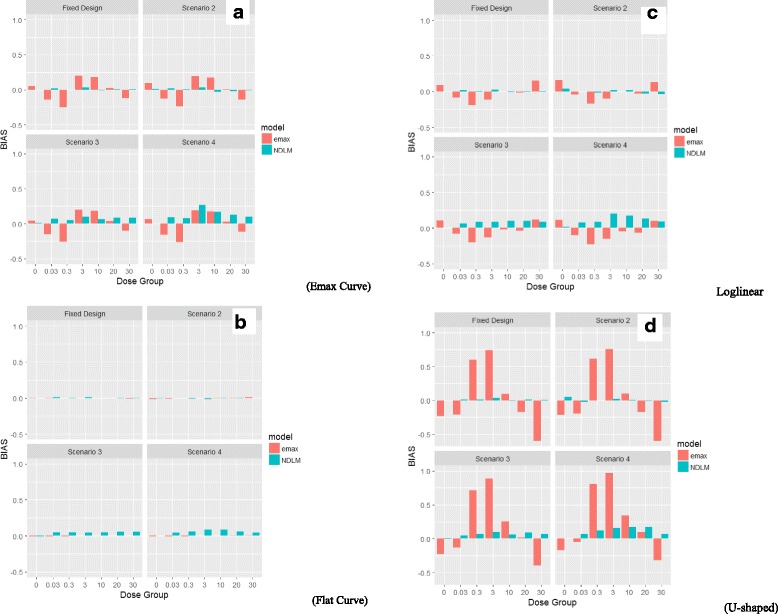
The statistical bias based on the fixed Design and design with adaptations. The statistical bias is based on each planned dose group (placebo, 0.03, 3, 10, 20, and 30 mg/kg) under four scenarios of design setting as fixed design, no adaptive (Scenario 2), half adaptive (Scenario 3) and fully adaptive (Scenario 4). The true dose responses follow dose profiles of **a**: *Emax* curve; **b**: flat curve; **c**: log linear curve and **d**: U-shaped curve

Under the fixed design and no adaptive allocation and assumption of true dose response as *Emax* like curve (Fig. 5a) or log linear (Fig. 5c) shape curve, there is less bias (absolute bias) of mean response at lower dose levels using the NDLM model in comparison to the Bayesian *Emax* model. The bias using *Emax* model is less if the true dose response data follow a placebo like response (Fig. 5b) than NDLM model and the absolute values of all bias are less than 0.02. If the true dose response curve is a U Shaped non-monotonic curve (Fig. 5d), the bias is much bigger at 0.3 mg/kg and 3 mg/kg if analysing using the *Emax* model (0.6510 in *Emax* model vs. -0.0062 at 0.3 mg/kg in the NDLM model; 0.7523 in *Emax* model vs. 0.0155 at 3 mg/kg in the NDLM model), since the *Emax* model makes the assumption of monotonic changes and still fits the line between the lowest dose and highest dose, ignoring the U-shaped response.

Under the half adaptive allocation design and the assumption of true dose response as an *Emax* like curve or log linear shape curve, similar to fixed design, there are less bias (absolute bias) of mean response at lower dose levels but more bias at 20 mg/kg using the NDLM model in comparison to the Bayesian *Emax* model. The individual bias from each dose level shows that *Emax* model tends to underestimate the dose response effect while NDLM tends to overestimate the effect in the mean response. The bias using *Emax* model is less if the true dose response data follow a placebo like response than NDLM model and the absolute values of all bias are less than 0.06. If the true dose response curve is a U-Shaped non-monotonic curve, the bias is much bigger at 0.3 mg/kg and 3 mg/kg if analysing using the *Emax* model (0.7182 in *Emax* model vs. 0.0656 at 0.3 mg/kg in the NDLM model; 0.8835 in *Emax* model vs. 0.0992 at 3 mg/kg in the NDLM model) for the same reason described earlier.

Under the fully adaptive allocation design and the assumption of the true dose response as an *Emax* like curve, the bias of the Bayesian *Emax* model and NDLM model is similar. The individual bias from each dose level shows that *Emax* model tends to underestimate the mean response effect at 0.03, 0.3 and 30 mg/kg while NDLM tends to overestimate the effect at 3 and 20 mg/kg in the mean response. The biases are also similar if the true dose response data follow a log linear curve and *Emax* model tends to underestimate the mean response while NDLM tends to overestimate the mean response. NDLM model also overestimate the mean response if the true response is placebo like curve. If the true dose response curve is a U-Shaped non-monotonic curve, the bias is much bigger at 0.3 mg/kg and 3 mg/kg if analysing using the *Emax* model (0.8013 in *Emax* model vs. 0.1170 at 0.3 mg/kg in the NDLM model; 0.9678 in *Emax* model vs. 0.1553 at 3 mg/kg in the NDLM model).

Amongst all the designs, a hybrid approach of half adaptive design with fixed allocation at 50% subjects before any adaptive allocation seems to have the most reasonable operating characteristics and will be considered to carry forward for GSK654321. To further explore the impact of the analysis methods additional simulations were undertaken to examine the impact of choice of informative priors but anchored in the single half adaptive design (S3). The results for the *Emax* model are given below in Table 4.

**Table 4:** Probability of success and failures at interim and final analysis with Bayesian *Emax* model with informative prior (β1 ~ N(−0.5, 1.2*1.2), β2 ~ N(−2.9, 1.2*1.2) and β3 ~ N(3, 2*2), β1, β2, and β3 are parameter estimates of E0, Emax and ED50 respectively) in the half adaptive design (Scenario 3)

True Dose Response	Bayesian *Emax* model					
	Early success	Early failure	Final success	Final failure	Total Success	Mean subjects
Placebo like flat Curve	0.00	0.13	0.08	0.79	8%	63
*Emax* Curve	0.87	0.00	0.13	0.00	100%	51
Log Linear Curve	0.71	0.00	0.28	0.01	99%	54
U-Shaped Curve	0.18	0.00	0.41	0.40	59%	61

The probability of success, as measure of posteriors probability of treatment effect (difference between treatment and placebo) greater than zero, increased in all dose response curves with 100%, 99, 59% success if the dose response follows a *Emax* model, Loglinear model and U-shaped curve. The type I error rate is inflated to 8% in *Emax* model with the informative prior. This inflated type I error rate would need to be communicated to the study team who may consider this to be too high a development risk.

Additional simulations for the NDLM model were performed to examine the impact of informative prior on the half adaptive design (S3) and are displayed below with two prior choices a) the evolution variance has prior of Inverse Gamma (IG) distribution (IG(0.5,0.5)) and b) IG(2,4).

The additional simulations seem to show that the NDLM model fitting is sensitive to the choice of evolution variance and the probably of success and type I error are impacted by the choice of priors such that with an informative prior, type I error was reduced to as low as 7% with little impact of the probability of success in other dose response curve. These considerations need to be weighed up by the study team. If the Type I error is important then the priors may be further investigated to reduce these to an acceptable level.

To compare the goodness of model fitting, deviance information criteria (DIC) results were calculated for both *Emax* and NDLM model based on dataset from single simulation in Half adaptive design. DIC was penalized for overfitting with additional parameters in the model. The DIC for NDLM model was 181.1 in comparison to 187.0 for *Emax* model, which further showed that there was no overfitting in NDLM model.

### Summary of model comparison: *Emax* model versus NDLM model

If dose response follows a monotonic response i.e. *Emax* or log linear curve, both Bayesian *Emax* and NDLM models have good operating characteristic in the probability of success at interim and final analysis. However, a Bayesian *Emax* model performs better with higher probability of success than NDLM model in all the scenarios.

If the dose effects change non-monotonically in a U-shaped dose response curve, the power measured as the probability of success of the Bayesian *Emax* model is 26% vs 92% using the NDLM model in fixed design, 24% vs 80% in No-adaptive design, 16% vs 88% in Half-Adaptive design and 24% vs 86% in Adaptive design. The NDLM model significantly improves the probability of success compared to the *Emax* model in all four design simulations.

Under the same decision criteria, the Type I error rates are elevated to 12% for half-adaptive or fully adaptive scenario and to 18% for a non-adaptive scenario when analysing using the NDLM model, while the type I error is generally under control below 5% using *Emax* model. An inflated Type I error rate signals that the NDLM model is over-sensitive and is thus inflating the number of false positive trials. When controlling Type I error, it was shown from ROC curves that the statistical power is 8–10% lowers in NDLM model if the dose response follows *Emax* or Loglinear curves but much better in case of U-shaped curve. Analysis of the NDLM model led to a significant increase in the statistical power of detecting the treatment difference, when the true dose response is non-monotonic, compared to the Bayesian *Emax* Model. The probability of success using NDLM model was similar regardless of which underlying true dose-response profile was assumed, but less sensitivity in the analysis of selecting the dose response of ED90 and an increase in the statistical bias, compared to the Bayesian *Emax* model. The Bayesian *Emax* model excelled with a higher probability of selecting ED90 and a smaller average sample size, when the true dose response followed *Emax* like curve, compared to NDLM model.

Though there were some variations, the bias is comparable if the true dose response follows a placebo like curve, *Emax* like curve, or log linear shape curve under the no adaptive allocation, half adaptive and adaptive scenarios. The bias for *Emax* is significantly increased if the true dose response is assumed to follow a U-shaped non-monotonic curve.

## Discussion

Due to the fact that the results for a PoC RA study of a drug in the same class followed a U-shaped dose response there was a wish to investigate if the analysis could be improved for a new compound in development. Of particular interest, in context with the development for GSK654321, the NDLM model was able to maintain the probability of success even in the case of a non-monotonic dose response.

We were conscious that the design of GSK654321 was driven by a single study for a lead compound, GSK123456, the analysis of which seemed to show a U-shaped dose response and the U-shaped dose response was deemed pharmacologically plausible [19]. Given the limitations of the NDLM model when the response is not U-shaped we decided to undertake further investigations of the U-shaped dose response in a literature review to assess the likelihood - based on the literature - of seeing this dose response relationship. It is shown that it is plausible to observe a U-shaped curve in the study with RA patients [21, 22]. Thomas et al. [6] showed that in the majority of cases the observed means could be well described using a Bayesian *Emax* model and *Emax* is one of the best models to estimate the dose response if data follows *Emax* curve, however, while biological exposure response relationships are often monotonic, down-turns of the clinical dose-response relationship at higher doses have been observed, one example in biologics development is the immunogenicity observed at high dose in the patients treating with biologicals. Therefore, we recommend to routinely consider a U-shaped dose-response model unless U-shaped profiles can be excluded with certainty at the trial design stage.

The work in this manuscript was inspired by the PoC design of the follow-on compound after the U-shaped curve was found in earlier clinical trial, which Bayesian *Emax* model was used. We aim to compare it with a more flexible NDLM model in the PoC design of the follow-on compound. Systematic literature search was conducted in the databases Google scholar, PubMED and web of science (WoS) and there was limited existing Literature in the comparison of *Emax* and NDLM model. Work by Jane Temple [23, 24] was deemed relevant but, within the parameters of the simulation undertaken by the authors although the research of Temple was of interest the work could not be generalised to the study being planned and described in this paper. This work demonstrated that both Bayesian NDLM model and *Emax* model detect a dose response well but Bayesian NDLM tends to have the highest power in the probability of detecting a clinical response than *Emax* model in the non-monotonical dose response.

It was also shown in the research of Temple that Bayesian NDLM tended to underestimate the response at lower doses, therefore resulting in higher doses being selected, however, our simulation showed a similar or better model fitting in Bayesian NDLM model than *Emax* model within the context of Phase 2a design. In addition, we found out that the adaptive design being proposed seemed to perform better with smaller average sample size but there was little difference in different allocation methods using NDLM model. These results agree with the finding in Temple [23, 24].

It has been reported that a Bayesian logistic model, especially with hierarchical longitudinal modelling with unbounded priors, often does not converge well [25, 26], posing a significant risk to dose escalation analysis. However, the NDLM model is a good alternative to the *Emax* model at the expense of pharmacological meaning in model parameters like maximal response *Emax* and ED50. This is to use an alternative, less complicated, modelling such as the linear model, power model etc. or a non-parametric model, such as the spline model or NDLM model. This will reduce the risk of non-convergence. A more Informative distribution on priors that constrain the parameter space to reasonable values would help the convergence for both models [27].

The main cause for concern with NDLM was the inflation of the Type I error. To minimise this problem, the decision criteria or informative prior may need to be adjusted to control the Type I error if the same decision rules are used in the comparison. After controlling for the type I error rate at 5%, the statistical powers of *Emax* model are ~8% higher than that of NDLM models in *Emax* and Log-linear dose responses, which was further supported by ROC results. The NDLM model works better when dose response follows U-shaped curve. Further work would be required therefore for any individual study to optimise the design characteristics. It is also acknowledged that NDLM model did not have high specificity in finding ED90 compared with the *Emax* model when the data follow *Emax* model.

It should be noted that the methods described in this paper were anchored in a single RA example with the simulations and results presented only applicable to this case study which motivated our work. This is of particular importance if different dose responses are anticipated or are of importance for an evaluation. Even for this case study there would be a need for further work once the study design has been finalised. In cases where a U-shaped curve is expected or there is potential physiological/pharmacological rationale of down-turn response, Bayesian NDLM model is generally recommended and this conclusion can be generalized to other case studies. In addition, our methods of evaluation in finding the best design could be generalised to other clinical trials to offer a solution to expedite drug development.

## Conclusion

An adaptive design, especially a half-adaptive design, is more a efficient design than a fixed design due to an increased chance of a dose being selected being the ED90 dose and due to the reduced s average sample size being use in the clinical trial. In most cases the Bayesian *Emax* model works effectively and efficiently, with low bias and good probability of success when there is a monotonic dose response. However, if there is a belief that the dose response could be non-monotonic based on prior knowledge as in our case study - where a compound in the same class seemed to have non-monotonic dose responses - then the NDLM is the superior model to assess the dose response. Within the parameters of the simulation the NDLM model was shown to be flexible with the ability to handle a wide variety of dose-responses, including monotonic and non-monotonic relationships.

## Additional file

Additional file 1: Supplemental materials provide additional results to supplement the main manuscript, including the two tables and two figures to discuss the proportional of doses being selected as ED90 and ROC curves in non-adaptive and fully adaptive scenarios.﻿ (DOCX 47 kb)

## Abbreviations

ASTIN: Acute stroke therapy by inhibition of neutrophils
CRP: C reactive protein
DAS28: Disease activity score based on 28 joints
ESR: Erythrocyte sedimentation rate
FACTs: Fixed and adaptive clinical trial simulator
MCMC: Markov chain ﻿Monte Carlo
NDLM: Normal dynamic linear model
PoC: Proof-of-concept
R&D: Research and development
RA: Rheumatoid ﻿A﻿rthritis
WoS: Web of Science
