# Supplementary material for: Design considerations and analysis planning of a phase 2a proof of concept study in rheumatoid arthritis in the presence of possible non-monotonicity
Source: BMC Med Res Methodol. 2017 Oct 2;17:149. doi: 10.1186/s12874-017-0416-3 (PMC5625783; doi:10.1186/s12874-017-0416-3)
Supplement: Additional file 1: — Supplemental materials provide additional results to supplement the main manuscript, including the two tables and two figures to discuss the proportional of doses being selected as ED90 and ROC curves in non-adaptive and fully adaptive scenarios.﻿ (DOCX 47 kb) [file 12874_2017_416_MOESM1_ESM.docx]

Table S1 Proportion of doses being selected as ED90 of Bayesian Emax and NDLM model at different dose response curves in the non-adaptive design settings (Scenario 2)

|  | **Dose Level (mg/kg)** | | | | | |
| --- | --- | --- | --- | --- | --- | --- |
|  | **0.03** | **0.3** | **3** | **10** | **20** | **30** |
| **Bayesian *Emax* Model** |  |  |  |  |  |  |
| Proportion of doses being selected as ED90 |  |  |  |  |  |  |
| Flat placebo like Curve * | 0% | 0% | 0% | 0% | 35% | 0% |
| *Emax* like Curve | 0% | 0% | 0% | 0% | 92% | 8% |
| Log Linear Curve | 0% | 0% | 0% | 0% | 83% | 16% |
| U Shape Curve * | 0% | 0% | 0% | 0% | 71% | 0% |
|  |  |  |  |  |  |  |
| **Bayesian NDLM Model** |  |  |  |  |  |  |
| Proportion of doses being selected as ED90 |  |  |  |  |  |  |
| Flat placebo like curve | 18% | 17% | 18% | 13% | 13% | 14% |
| *Emax* like Curve | 1% | 2% | 15% | 24% | 33% | 25% |
| Log Linear Curve | 2% | 3% | 6% | 13% | 28% | 48% |
| U Shape Curve | 4% | 38% | 44% | 7% | 6% | 2% |
| * ED90 is missing where the maximum dose was not estimated correctly. | | | | | | |

Table S2 Proportion of doses being selected as ED90 of Bayesian Emax and NDLM model at different dose response curves in the fully adaptive design settings (Scenario 4).

|  | **Dose Level (mg/kg)** | | | | | | |
| --- | --- | --- | --- | --- | --- | --- | --- |
|  | **0.03** | **0.3** | | **3** | **10** | **20** | **30** |
| **Bayesian *Emax* Model** |  |  | |  |  |  |  |
| Proportion of doses being selected as ED90 |  |  | |  |  |  |  |
| Flat placebo like Curve * | 0% | 0% | | 0% | 0% | 36% | 1% |
| *Emax* like Curve | 0% | 0% | | 0% | 0% | 92% | 8% |
| Log Linear Curve | 0% | 0% | | 0% | 0% | 91% | 8% |
| U Shape Curve * | 0% | 0% | | 0% | 0% | 59% | 0% |
|  |  |  | |  |  |  |  |
| **Bayesian NDLM Model** |  |  | |  |  |  |  |
| Proportion of doses being selected as ED90 |  |  | |  |  |  |  |
| Flat placebo like curve | 15% | 15% | | 16% | 16% | 11% | 11% |
| *Emax* like Curve | 0% | 2% | | 14% | 24% | 34% | 27% |
| Log Linear Curve | 1% | 2% | | 6% | 16% | 26% | 49% |
| U Shape Curve | 1% | 39% | | 52% | 6% | 1% | 0% |
| * ED90 is missing where the maximum dose was not estimated correctly. | | |  |  |  |  |  |

B

A

C

Figure S1 ROC curves display the true positive rate (statistical power) and false positive rate for Bayesian *Emax* (red) and NDLM model (blue) under non adaptive design (Scenario 2) at dose response following A) U-Shaped, B) Emax or C) Loglinear curves

B

A

C

Figure S2 ROC curve display the true positive rate (statistical power) and false positive rate for Bayesian *Emax* (red) and NDLM model (blue) under fully adaptive design (Scenario 4) with the dose response following A) U-Shaped, B) Emax or C) Loglinear curves
